# Gender difference in the association between composite dietary antioxidant index and all-cause mortality

**DOI:** 10.3389/fnut.2025.1523171

**Published:** 2025-03-04

**Authors:** Lanzhi Duan, Rui Zeng, Jiang Wang, Sisi Hu, Weiye Wang

**Affiliations:** ^1^Department of Public Health, School of Basic Medical Sciences, Jinggangshan University, Ji'an, Jiangxi, China; ^2^School of Clinical Medicine, Jinggangshan University, Ji'an, Jiangxi, China; ^3^The Personnel Department, Jinggangshan University, Ji'an, Jiangxi, China; ^4^Jiangxi Province Key Laboratory of Organ Development and Epigenetics, Clinical Medical Research Center, Affiliated Hospital of Jinggangshan University, Medical Department of Jinggangshan University, Ji'an, Jiangxi, China

**Keywords:** all-cause mortality, antioxidant index, composite dietary, gender difference, NHANES

## Abstract

**Background:**

Existing studies on the association between the composite dietary antioxidant index (CDAI) and all-cause mortality are controversial. We aimed to analyze the association of CDAI with all-cause mortality, and determine the influence of gender on this association.

**Methods:**

The data of adult participants (age ≥ 18) from the National Health and Nutrition Examination Survey (NHANES) cycles spanning 2001 to 2018 were analyzed. The NHANES-issued identifiers for participants enabled the linkage of data from the NHANES Public Use Linked Mortality File.

**Results:**

The study encompassed a sample of 15,651 individuals. The mean CDAI was 0.52 ± 6.06. The restricted cubic spline revealed that the hazard ratio (HR) of all-cause mortality decreased significantly with increasing CDAI. However, this negative association existed only when the CDAI was less than 5. Multivariate Cox regression analysis showed that compared to the first CDAI quartile, the HR of all-cause mortality was significantly decreased in the third and fourth quartiles (both *p* < 0.001), and the *p*-value of the trend test was <0.001. In the subgroup analysis, a notably strong negative association between CDAI and the risk of all-cause mortality was only observed in men (*p* for interaction <0.001).

**Conclusion:**

Higher CDAI is associated with a reduced risk of all-cause mortality exclusively in adult males, underscoring the substantial influence of gender on this relationship.

## Introduction

Excessive reactive oxygen species (ROS) production leads to oxidative stress, which surpasses the body’s antioxidant capabilities, resulting in cellular and DNA damage (1). Persistent damage from ROS can elevate the likelihood of chronic non-communicable diseases, notably cancer and cardiovascular diseases (CVDs), which are major contributors to global mortality rates. This highlights the importance of managing oxidative stress to reduce these health risks (2). Chronic inflammation plays a pivotal role in the initiation and advancement of several health disorders, such as obesity, CVDs, diabetes, and cancer. It is also linked to an increased risk of all-cause mortality (3, 4).

Dietary antioxidants, such as vitamins A, C and E, zinc, selenium, and carotenoids, can combat non-communicable diseases (NCDs) by neutralizing free radicals and reducing oxidative stress (5). In addition, an antioxidant-rich diet has been demonstrated to substantially decrease levels of inflammation, leading to improved conditions of NCDs and a lowered risk of mortality (6–8). The composite dietary antioxidant index (CDAI) is increasingly being recognized as a potential tool for mitigating oxidative stress and chronic inflammation (9). CDAI is calculated by summing the standardized intake values of six antioxidants: vitamin A, vitamin C, vitamin E, zinc, selenium, and total carotenoids (10, 11). Studies have demonstrated that CDAI is significantly associated with various health outcomes, including delaying aging, reducing the risk of chronic diseases, and improving metabolic health (12–15). As a comprehensive index, CDAI can evaluate the intake of multiple antioxidant substances in an individual’s diet more comprehensively and accurately reflect the overall dietary impact on oxidative stress, rather than focusing solely on single antioxidants. Additionally, CDAI provides a scientific basis for dietary interventions by identifying individuals with insufficient antioxidant intake, thereby promoting healthier dietary practices. In daily life, one can obtain vitamin A and carotenoids from dark green vegetables like spinach and kale, as well as orange vegetables and fruits such as carrots, sweet potatoes, mangoes, tomatoes, and sweet peppers (16). Nuts and seeds, including almonds, sunflower seeds, and Brazil nuts, are rich sources of zinc, vitamin E, and selenium (17). Animal-based foods such as eggs, oysters, and shrimp provide vitamins A and E (18). Citrus fruits like oranges and grapefruits are excellent sources of vitamin C and carotenoids (19). Whole grains and legumes, such as quinoa and lentils, contribute to the intake of zinc and selenium (20).

The association of CDAI with all-cause mortality remains an active area of research. Previous studies conducted in the USA have shown that CDAI is negatively correlated to the risk of all-cause mortality in adults, and with the risk of type 2 diabetes and osteoarthritis among the middle-aged and older adults (13, 21, 22). However, a large cohort study from the UK gave conflicting evidence, and subgroup analysis showed that CDAI was not significantly associated with either non-cancer or cancer-related deaths (23). Evidence from cross-sectional studies also supports this finding (10). Overall, the association between CDAI and all-cause mortality remains controversial, and studies on potential effect modification roles are lacking.

Earlier studies have primarily examined the relationship between CDAI and all-cause mortality but have overlooked the impact of gender differences on this association. Given the significant role that sex differences play in various physiological and pathological processes, our study used the National Health and Nutrition Examination Survey (NHANES) database to analyze the association between CDAI and all-cause mortality, and determine the influence of gender on this association.

## Methods

### Data sources and participants

The data from nine 2-year cycles (2001–2018) of the NHANES were used. This survey used a stratified, multi-stage probabilistic design to obtain a nationally representative sample of approximately 5,000 individuals per year to assess the nutritional and health status of the population^1^. All participants had provided written informed consent.

**Figure 1:**
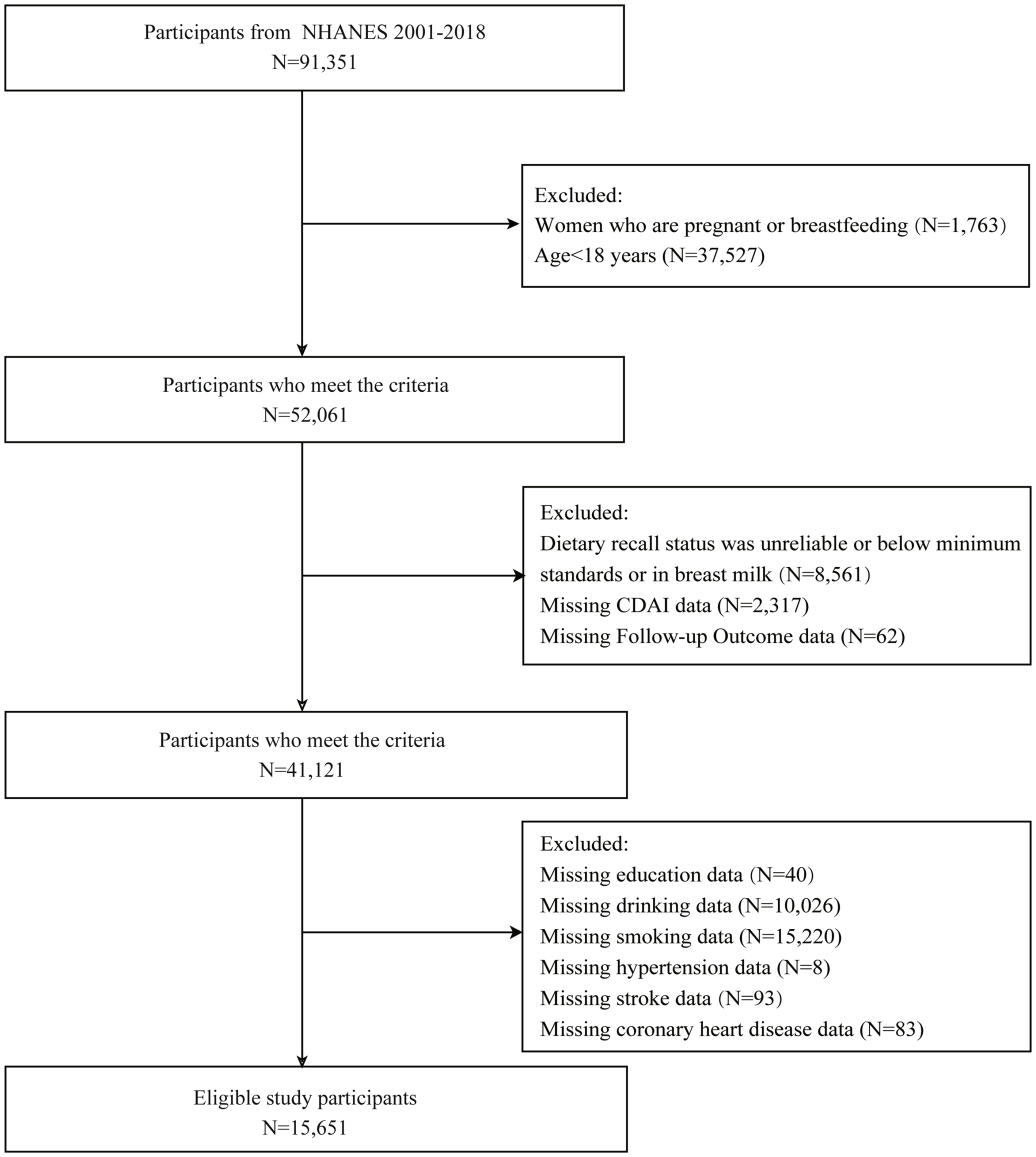
Flowchart of the study population.

The inclusion criterion for the participants was age ≥ 18 years. The exclusion criteria were as follows: (1) pregnant or lactating women; (2) unreliable dietary recall status, or being below the minimum standard, or breastfeeding; (3) missing CDAI data; (4) missing follow-up data; (5) missing data on education, alcohol use, smoking, hypertension, stroke, or coronary heart disease. A total of 15,651 study subjects were ultimately enrolled (Figure 1).

### Composite dietary antioxidant index

We used dietary information recorded in NHANES to calculate CDAI. The 24-h dietary recall interview documented information of six dietary antioxidants, including vitamin A, C and E, zinc, selenium, and total carotenoids (α-carotene, β-carotene, β-cryptoxanthin, lycopene, lutein, and zeaxanthin) (10, 11). Each participant was interviewed twice for the dietary intake, initially at the baseline health check and then over the next 3–10 days. The dietary recall data from both occasions were assessed for reliability and the mean daily intake was calculated. The consumption of each antioxidant was normalized by first subtracting the average value and then dividing by the standard deviation. The resulting normalized values were summed to calculate the CDAI (24).

### All-cause mortality

To accurately assess all-cause mortality in the follow-up population, the NHANES serial numbers were used to link the data extracted from the NHANES Public Use Linked Mortality File on December 31, 2019 with the NHANES database. The relevant causes of death were defined using the 10th Revision of the National Statistical Classification of Diseases (ICD-10), and deaths from any cause were considered to be all-cause mortality (25).

### Covariates

Age, gender, race, and education level were self-reported by participants at the baseline health examination. Smoking status was derived from self-reported responses to the question “Do you currently smoke?.” The responses were categorized as “not at all,” “some days” and “every day.” The frequency of alcohol consumption in the previous year was also categorized as “not at all,” “some days” and “every day.” Diabetes mellitus was defined as positive diagnosis by a physician, current usage of glucose-lowering medication, fasting blood glucose ≥126 mg/dL, and glycated hemoglobin ≥6.5% (26). Hypertension was defined as diagnosis of high blood pressure by a physician, current usage of anti-hypertensive medications, systolic blood pressure ≥ 130 mmHg, and a diastolic blood pressure ≥ 80 mmHg (27). Stroke and coronary heart disease were defined according to clinical diagnosis (28).

### Statistical analysis

The mean values along with their standard deviations (denoted as mean ± SD) were used to illustrate the numerical data, whereas the categorical data was depicted using percentage values. One-way ANOVA was used to compare population means and Pearson’s chi-square test was used to compare population rates. The shape of the association between CDAI and all-cause mortality was described by a 5-node (5.00th, 27.50th, 50.00th, 72.50th, 95.00th) restricted natural cubic splines model, and the hazard ratio (HR) and significance of the relationship between CDAI and all-cause mortality were further analyzed by multivariate Cox regression models. We also performed sensitivity analyses according to CDAI level tripartite grouping.

The survival rates of male and female participants grouped in the different CDAI quartiles were analyzed by plotting Kaplan–Meier curves. The potential effect of gender on the association between CDAI and all-cause mortality was determined by multiplicative interaction analyses. R software version 4.4.1 was used for all statistical analyses, and *p*-value <0.05 was considered statistically significant.

## Results

### Baseline characteristics of study participants

A total of 15,651 participants were included in the final data analysis. 9,427 (60.23%) were men, with a mean BMI of 29.00 ± 6.72. Some day drinker were 11,386 (72.75%), while some day smoker were 1,318 (8.42%). 2,846 (18.18%) were diabetes, 8,413 (53.75%) were hypertensive, 973 (6.22%) were coronary heart disease, 754 (4.82%) were Stroke, and 3,030 (19.36%) died of any cause. The mean values of vitamin A and selenium were 0.61 ± 0.81 (mg) and 0.11 ± 0.06 (mg), respectively, the mean values of vitamin C, vitamin E and zinc were 81.11 ± 94.44 (mg); 7.45 ± 6.04 (mg); 11.56 ± 9.20 (mg), respectively. The average CDAI of the study population was 0.52 ± 6.06. Participants in the highest CDAI quartile had fewer women, lower BMI, lower diabetes, hypertension, stroke, and all-cause mortality. The baseline data of the patients in the different CDAI quartiles are summarized in Table 1.

**Table 1:** Baseline characteristics of study participants.

Characteristics	Total *n* = 15,651	CDAI				*p*-value
		Q1 (<−3.37) *n* = 3,913	Q2 (−3.37 ~ −0.81) *n* = 3,912	Q3 (−0.81 ~ 2.83) *n* = 3,913	Q4 (>2.83) *n* = 3,913	
Age (years)	52.38 ± 17.23	51.27 ± 17.15	52.67 ± 17.47	53.18 ± 17.23	52.42 ± 17.01	<0.001
Gender						<0.001
Male	9,427 (60.2)	1954 (49.9)	2,264 (57.9)	2,525 (64.5)	2,684 (68.6)	
Female	6,224 (39.8)	1959 (50.1)	1,648 (42.1)	1,388 (35.5)	1,229 (31.4)	
BMI (kg/m^2^)	29.00 ± 6.72	29.10 ± 6.89	29.15 ± 6.74	29.08 ± 6.71	28.66 ± 6.52	<0.001
Race						<0.001
Mexican American	2,131 (13.6)	536 (13.7)	550 (14.1)	501 (12.8)	544 (13.9)	
Non-Hispanic Black	3,082 (19.7)	929 (23.7)	752 (19.2)	707 (18.1)	694 (17.7)	
Non-Hispanic White	8,482 (54.2)	1967 (50.3)	2,136 (54.6)	2,213 (56.6)	2,166 (55.4)	
Other Hispanic	1,044 (6.7)	283 (7.2)	257 (6.6)	263 (6.7)	241 (6.2)	
Others	912 (5.8)	198 (5.1)	217 (5.5)	229 (5.9)	268 (6.8)	
Education						<0.001
Less than high school	2,159 (13.8)	735 (18.8)	523 (13.4)	484 (12.4)	417 (10.7)	
High school or equivalent	6,173 (39.4)	1752 (44.8)	1,649 (42.2)	1,431 (36.6)	1,341 (34.3)	
College or above	7,319 (46.8)	1,426 (36.4)	1740 (44.5)	1998 (51.1)	2,155 (551)	
Smoking status						<0.001
Not at all	8,649 (55.3)	1756 (44.9)	2090 (53.4)	2,332 (59.6)	2,471 (63.2)	
Some days	1,318 (8.4)	326 (8.3)	317 (8.1)	317 (8.1)	358 (9.1)	
Every day	5,684 (36.3)	1831 (46.8)	1,505 (38.5)	1,264 (32.3)	1,084 (27.7)	
Drinking status						<0.001
Not at all	4,216 (26.9)	1,186 (30.3)	1,074 (27.5)	1,035 (26.5)	921 (23.5)	
Some days	11,386 (72.8)	2,714 (69.4)	2,823 (72.2)	2,869 (73.3)	2,980 (76.2)	
Every day	49 (0.3)	13 (0.3)	15 (0.4)	9 (0.2)	12 (0.3)	
Diabetes						0.151
No	12,805 (81.8)	3,157 (80.7)	3,211 (82.1)	3,204 (81.9)	3,233 (82.6)	
Yes	2,846 (18.2)	756 (19.3)	701 (17.9)	709 (18.1)	680 (17.4)	
Hypertension						0.271
No	7,238 (46.2)	1764 (45.1)	1811 (46.3)	1812 (46.3)	1851 (47.3)	
Yes	8,413 (53.8)	2,149 (54.9)	2,101 (53.7)	2,101 (53.7)	2062 (52.7)	
Coronary heart disease						0.579
No	14,678 (93.8)	3,682 (94.1)	3,669 (93.8)	3,653 (93.4)	3,674 (93.9)	
Yes	973 (6.2)	231 (5.9)	243 (6.2)	260 (6.6)	239 (6.1)	
Stroke						<0.001
No	12,621 (80.6)	3,106 (79.4)	3,091 (79.0)	3,201 (81.8)	3,223 (82.4)	
Yes	3,030 (19.4)	807 (20.6)	821 (21.0)	712 (18.2)	690 (17.6)	

CDAI, composite dietary antioxidant index. BMI, body mass index.

### Hazard ratio ratios for incident all-cause mortality

Multivariate Cox regression analysis was performed to quantify the association between CDAI and the risk of all-cause mortality. Using the first quartile of CDAI as the reference group, we observed a significant decrease in the HR of all-cause mortality in the third and fourth quartiles of the study participants (both *p* < 0.05). In addition, the *p*-value of the trend test was less than 0.05. The data are summarized in Table 2. Sensitivity analysis showed that compared with the low (−8.375 ~ −2.5033) group, the HR and *p* values of the middle (−2.5029 ~ 1.3304) and high (1.3305 ~ 195.5575) groups were 0.88 (0.81, 0.96), *p* = 0.006; 0.81 (0.74, 0.89), *p* < 0.001, and the trend test *p*-value was <0.001, as shown in Supplementary Table 1.

**Table 2:** Association of CDAI and all-cause mortality.

CDAI	All-cause mortality	Unadjusted	Model I	Model II	Model III
Q1	807 (20.6)	1.00 (1.00, 1.00)	1.00 (1.00, 1.00)	1.00 (1.00, 1.00)	1.00 (1.00, 1.00)
Q2	821 (21.0)	1.03 (0.94, 1.14)	0.88 (0.79, 0.97)^*^	0.93 (0.85, 1.03)	0.95 (0.86, 1.05)
Q3	712 (18.2)	0.87 (0.78, 0.96)^*^	0.70 (0.64, 0.78)^*^	0.80 (0.72, 0.88)^*^	0.81 (0.73, 0.89)^*^
Q4	690 (17.6)	0.81 (0.73, 0.90)^*^	0.68 (0.61, 0.75)^*^	0.80 (0.72, 0.89)^*^	0.81 (0.73, 0.90)^*^
*p* for trend		<0.001	<0.001	<0.001	<0.001

Model I was adjusted for age, gender and ethnicity. Model II was additionally adjusted for education level, smoking status and drinking status on the basis of Model I. Model III was additionally adjusted for diabetes, hypertension, stroke and coronary heart disease on the basis of Model II. ^*^p < 0.01.

**Figure 2:**
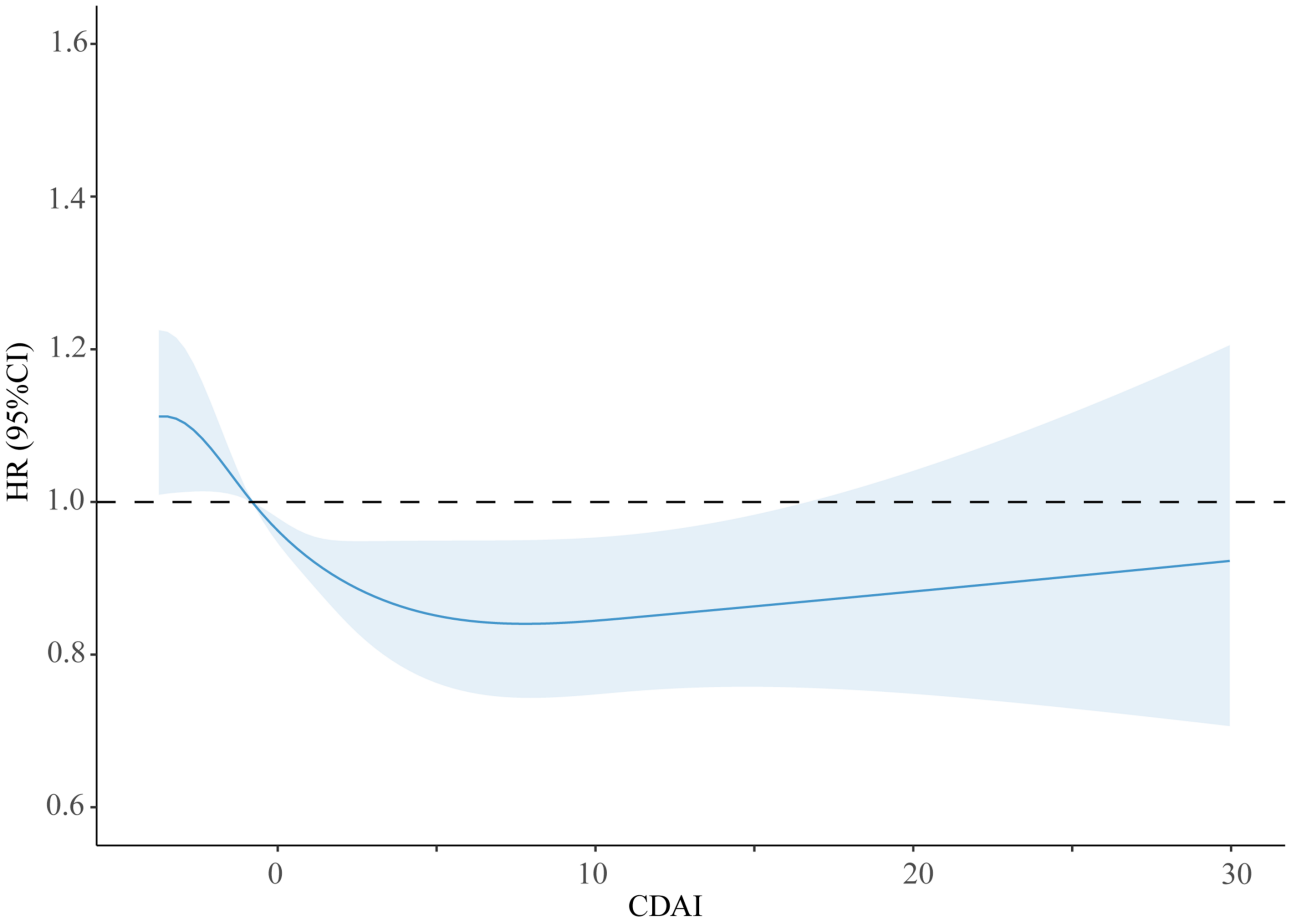
Restricted cubic splines of the association between CDAI and the risk of all-cause mortality.

Restricted natural cubic splines showed a gradual decrease in the HR of all-cause mortality with the increase in CDAI. However, mortality due to all causes was not reduced by the CDAI at values higher than 5, indicating a saturation effect (see Figure 2).

### Effect modification of gender on the association

**Figure 3:**
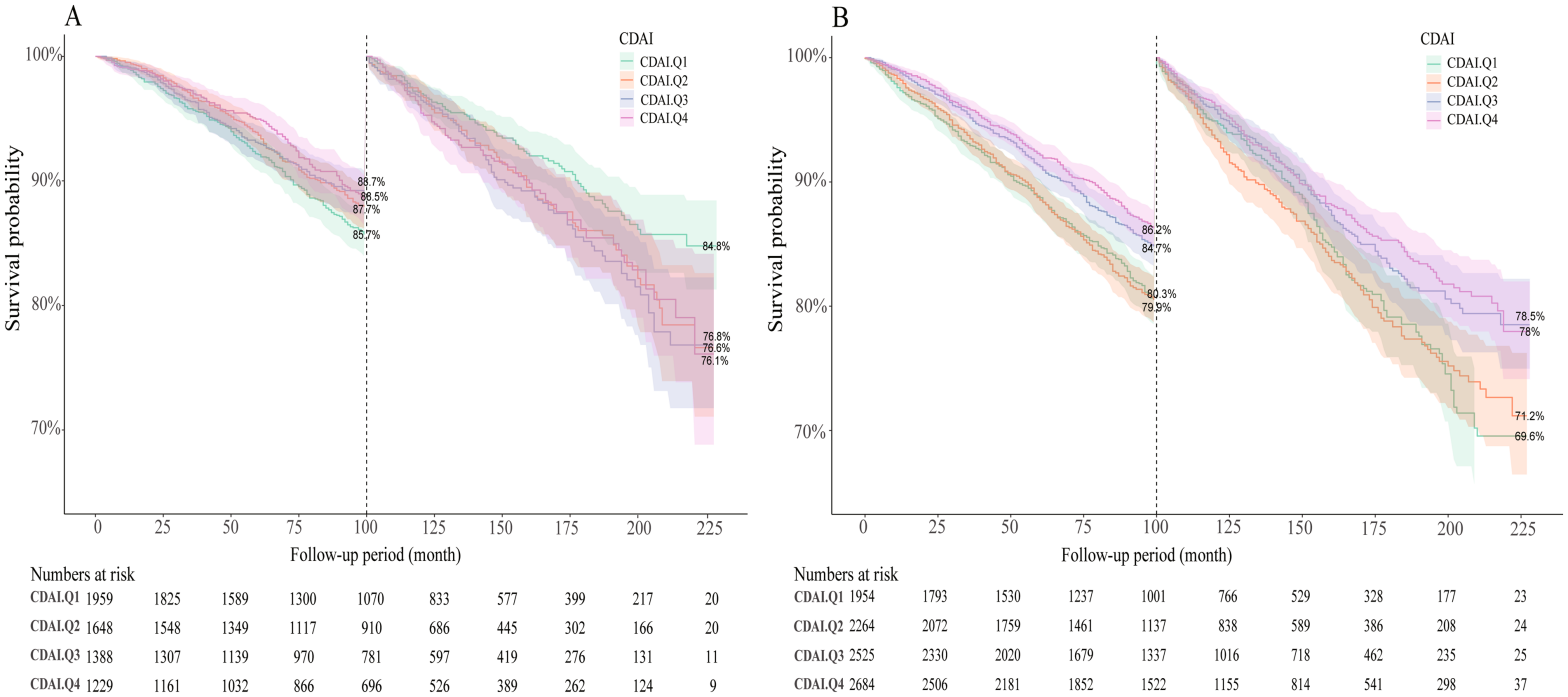
Kaplan–Meier survival curves showing cumulative probability of all-cause mortality in the CDAI quartile groups among **(A)** female and **(B)** male participants.

Subgroup analysis was also performed to explore the potential impact of gender on the association. As shown in Table 3, women were not significantly associated with CDAI and all-cause mortality. Sensitivity analyses yielded similar results, the relationship was only observed in men (Supplementary Table 2). Furthermore, we also observed a multiplication interaction (*p*-value for interaction <0.001). Consistent with this, the survival probability was higher in the third and fourth quartile groups of CDAI among males, and the log-rank test showed a statistically significant difference (*p* < 0.001). However, no significant difference was observed in the survival probabilities of the different quartile groups among females (*p* = 0.940). The data is summarized in Figure 3.

**Table 3:** Association between CDAI and all-cause mortality stratified by gender.

Gender	*N*	CDAI Q2		CDAI Q3		CDAI Q4		*p*_interaction_^*^
		*HR* (95% *CI*)	*p*	*HR* (95% *CI*)	*p*	*HR* (95% *CI*)	*p*	
Male	9,427	0.95 (0.84, 1.08)	0.455	0.76 (0.67, 0.87)	<0.001	0.79 (0.69, 0.90)	0.001	<0.001
Female	6,224	0.95 (0.80, 1.12)	0.529	0.93 (0.78, 1.12)	0.455	0.84 (0.70, 1.01)	0.069	

*p*_interaction_^*^, *p*-value for interaction. All models were adjusted for age, ethnicity, education level, smoking status, drinking status, diabetes, hypertension, stroke and coronary heart disease.

## Discussion

We found a negative association between CDAI and the risk of all-cause mortality in the adult population of USA. However, the association was not linear, and CDAI >5 did not cause a reduction in the risk of all-cause mortality. Furthermore, we observed this negative relationship only in men, indicating a significant impact of gender.

Previous studies have also shown a significant negative association between CDAI and all-cause mortality (13, 21, 29). However, a study based on UK Biobank data showed that antioxidant supplementation did not have a significant impact on cancer or non-cancer deaths (23). In addition, CDAI less than −0.19 was significantly associated with all-cause mortality in 45-year-olds in the United States (22). Likewise, CDAI demonstrated a protective association with CVD-related mortality (13). CDAI has been shown to have no significant association with all-cause mortality in certain specific populations, for example, patients with metabolic syndrome (MetS) (10). However, the applicability of this association in other specific populations remains to be explored in further studies. The inconsistencies among these studies may be due to the different characteristics of the populations and the differences in covariates. Our findings are in line with most previous studies.

CDAI and all-cause mortality are linked by specific biological mechanisms, but the mechanisms are not fully understood, although some studies have put forth several hypotheses. Normal antioxidant defense mechanisms in the body result in a disrupted balance between the two due to excessive production of free radicals, which eventually manifest as oxidative stress. Given that antioxidants slow down the aging process by reducing free radical damage to cells (30), the results of several studies indicate that high CDAIs are associated with greater longevity and reduced death rates from all causes. Damage to cells by free radicals resulting from oxidative stress can be relieved by the ingestion of foods rich in antioxidants (31). Chronic inflammation also plays an important role in the development and progression of numerous diseases, such as CVDs, cancer, and diabetes, and is also strongly associated with an elevated risk of all-cause mortality (5, 32). The food can significantly reduce the levels of inflammatory markers and mitigate the symptoms of chronic diseases, resulting in lower mortality risk (33–35). The DNA repair pathway is another potential mechanistic link between CDAI and mortality risk. Antioxidants protect DNA from free radical damage and repair DNA lesions, which is able to prevent DNA related diseases to some extent (36). Diabetes- and CVD-related deaths are important components of all-cause mortality. CDAI may reduce the risk of type 2 diabetes by improving insulin resistance and glucose metabolism (37). CVDs, although not directly classified as metabolic diseases, can be caused or exacerbated by metabolic abnormalities. Increasing the intake of antioxidants can reduce the risk of CVD-related deaths by regulating plasma REDOX status and neutralizing the reactive oxygen and nitrogen species (38). Notably, the association between CDAI and risk of all-cause mortality in the present study was only demonstrated in adults with CDAI <5, however, the exact dose that does not produce any promotion of oxidation is unknown, and the exact mechanism remains to be elucidated.

The significant negative association between CDAI and all-cause mortality was only observed in men, which was also reflected in the survival analysis. Furthermore, there was a significant multiplicative interaction. This is the first study to demonstrate that gender influences CDAI’s association with all-cause mortality. The most reasonable explanation is the differences in the levels of sex hormones between men and women, which may influence metabolism and antioxidant action. For example, estrogen is known to lower ROS production and oxidative stress by activating antioxidant pathways downstream of its specific receptors (estrogen receptors alpha and beta), which may reduce the benefit of dietary antioxidants on the risk of all-cause mortality (39, 40). Second, All-cause mortality is a significant long-term prognostic event for CVDs, and it is associated with a higher incidence of cardiovascular events in men compared to women (41). Given the substantial efficacy of CDAI in reducing deaths due to cardiovascular disease, this may be another reason why CDAI have a stronger protective effect in men (14).

This study presents several significant clinical implications. First, we observed a potential reduction in the risk of all-cause mortality among men associated with dietary antioxidant intake, highlighting the importance of gender differences in health interventions. The findings suggest that men should prioritize the consumption of foods rich in vitamins A, C, and E, as well as selenium and zinc (such as green vegetables, nuts, fish, etc.), to mitigate their risk of all-cause mortality. Instead, health interventions aimed at female populations should formulate comprehensive strategies that do not depend exclusively on diets rich in antioxidants. Second, the gender specificity inherent in CDAI may facilitate the establishment of mortality risk assessment models. Future studies could effectively identify individuals at high risk for death through CDAI and incorporate it into male health examination programs alongside other clinical indicators for a thorough evaluation. Finally, given that comorbidity is a recognized risk factor for all-cause mortality among older adults, it is advisable for clinicians to recommend an appropriate dietary regimen based on CDAI for male patients suffering from chronic conditions such as hypertension, hyperlipidemia, CVDs, and others. Future research should focus on conducting further clinical trials and mechanism-related investigations to develop and refine actionable public health strategies aimed at addressing the challenges posed by an aging population; thereby effectively extending healthy life expectancy.

We included a large number of covariables in the multifactor analysis to minimize any confounding bias. Furthermore, NHANES has a standardized method of collecting analytical data, along with strict quality control measures to ensure accuracy and reliability. Therefore, the information bias in this study was controlled within a reasonable range. Finally, the NHANES data is derived from a large sample that is representative of the population and can accurately reflect the health status.

There are some limitations in this study. First, the CDAI was derived from 24-h dietary recall interviews. Yet, the potential for recall bias and variability in daily dietary habits could introduce some imprecision into the findings. Second, it is unclear whether the dietary intake data collected at baseline changed during subsequent follow-up, which may have contributed to some information bias regarding CDAI. Third, although we included many confounding factors in the statistical analysis, we did not consider other compounds with antioxidant potential (e.g., phenolics and flavonoids), nor did we consider data on the consumption of supplements and related medications, due to a lack of data in the NHANES database. Fourth, this study was unable to further explore the independent effects of the six dietary antioxidants in reducing all-cause mortality due to a lack of relevant data as well as technical reasons. Finally, this study is only representative of U.S. adults, and it is necessary to verify the generalizability of the findings across culturally and geographically diverse populations.

## Conclusion

In conclusion, CDAI showed a significant negative association with the risk of all-cause mortality. However, the protective effect of CDAI was not observed when it exceeded 5. In addition, CDAI levels were negatively related to all-cause mortality risk only in men and not in women when gender was a modifier.

## Data Availability

Publicly available datasets were analyzed in this study. This data can be found at: https://www.cdc.gov/nchs/nhanes/.

## Footnotes

^1^ https://wwwn.cdc.gov/nchs/nhanes/Default.aspx
