# Supplementary material for: Gender difference in the association between composite dietary antioxidant index and all-cause mortality
Source: Front Nutr. 2025 Mar 4;12:1523171. doi: 10.3389/fnut.2025.1523171 (PMC11913696; doi:10.3389/fnut.2025.1523171)
Supplement: Supplementary file 1 [file Table_1.docx]

|  | Model Ⅰ | Model Ⅱ | Model Ⅲ | Model Ⅳ |
| --- | --- | --- | --- | --- |
| Low (-8.375~-2.5033) | 1 | 1 | 1 | 1 |
| Middle (-2.5029~1.3304) | 0.92 (0.85, 1.01) 0.068 | 0.81 (0.74, 0.88) <0.001 | 0.88 (0.80, 0.96) 0.003 | 0.88 (0.81, 0.96) 0.006 |
| High (1.3305~195.5575) | 0.80 (0.73, 0.87) <0.001 | 0.70 (0.64, 0.76) <0.001 | 0.80 (0.73, 0.88) <0.001 | 0.81 (0.74, 0.89) <0.001 |
| P for trend | <0.001 | <0.001 | <0.001 | <0.001 |

Supplementary table 1 | Hazard ratios of all-cause mortality by CDAI levels (three groups) among adults in NHANES 2001–2018

Model Ⅰ: Unadjusted. Model Ⅱ: Adjust for age, gender, ethnicity. Mode Ⅲ: Adjust for age, gender, ethnicity, education, smoking status, drinking status. Mode Ⅳ: Adjust for age, gender, ethnicity, education, smoking status, drinking status, diabetes, hypertension, stroke, coronary heart disease.

Supplementary table 2 | Subgroup analysis of composite dietary antioxidant index and all-cause mortality in NHANES 2001-2018 participants

|  | Composite dietary antioxidant index | | | |
| --- | --- | --- | --- | --- |
|  | N | Low | Middle | High |
| Age, years |  |  |  |  |
| ＜60 | 9,539 | 1 | 0.89 (0.74, 1.07) 0.223 | 0.94 (0.78, 1.14) 0.543 |
| ≥60 | 6,112 | 1 | 0.91 (0.82, 1.01) 0.063 | 0.79 (0.71, 0.88) <0.001 |
| Gender |  |  |  |  |
| Male | 9,427 | 1 | 0.89 (0.80, 0.99) 0.038 | 0.79 (0.70, 0.89) <0.001 |
| Female | 6,224 | 1 | 0.91 (0.78, 1.06) 0.231 | 0.88 (0.75, 1.03) 0.113 |
| Race |  |  |  |  |
| Mexican American | 2,131 | 1 | 1.23 (0.92, 1.64) 0.159 | 0.80 (0.56, 1.13) 0.200 |
| Other Hispanic | 1,044 | 1 | 1.14 (0.70, 1.88) 0.592 | 1.08 (0.60, 1.93) 0.807 |
| Non-Hispanic White | 8,482 | 1 | 0.85 (0.76, 0.95) 0.004 | 0.80 (0.71, 0.89) <0.001 |
| Non-Hispanic Black | 3,082 | 1 | 0.86 (0.71, 1.06) 0.156 | 0.86 (0.70, 1.05) 0.134 |
| Other Race | 912 | 1 | 0.97 (0.55, 1.69) 0.907 | 0.85 (0.45, 1.63) 0.625 |
| Education |  |  |  |  |
| Less than high school | 2,159 | 1 | 0.98 (0.82, 1.17) 0.823 | 0.87 (0.71, 1.06) 0.176 |
| High School Grad/GED or Equivalent | 6,173 | 1 | 0.87 (0.76, 1.01) 0.060 | 0.82 (0.71, 0.95) 0.008 |
| College Graduate or above | 7,319 | 1 | 0.88 (0.75, 1.02) 0.087 | 0.79 (0.68, 0.92) 0.002 |
| BMI*, kg/m² |  |  |  |  |
| ＜25 | 4,422 | 1 | 0.88 (0.75, 1.03) 0.120 | 0.81 (0.68, 0.96) 0.014 |
| ≥25 | 11,006 | 1 | 0.88 (0.79, 0.98) 0.025 | 0.80 (0.72, 0.90) <0.001 |
| Drinking status |  |  |  |  |
| Every day | 49 | 1 | 0.006 (0.0015, 0.0268) <0.001 | 0.005 (0.0012, 0.0174) <0.001 |
| Some days | 11,386 | 1 | 0.838 (0.7434, 0.9438) 0.004 | 0.794 (0.7039, 0.8962) <0.001 |
| Not at all | 4,216 | 1 | 0.975 (0.8509, 1.1168) 0.713 | 0.832 (0.7166, 0.9661) 0.016 |
| Smoking status |  |  |  |  |
| Every day | 5,684 | 1 | 0.83 (0.71, 0.98) 0.023 | 0.81 (0.69, 0.97) 0.019 |
| Some days | 1,318 | 1 | 1.09 (0.68, 1.75) 0.730 | 1.19 (0.73, 1.94) 0.490 |
| Not at all | 8,649 | 1 | 0.93 (0.83, 1.04) 0.188 | 0.82 (0.73, 0.92) 0.001 |
| Diabetes |  |  |  |  |
| No | 12,805 | 1 | 0.89 (0.80, 0.99) 0.026 | 0.79 (0.70, 0.88) <0.001 |
| Yes | 2,846 | 1 | 0.94 (0.79, 1.11) 0.434 | 0.90 (0.76, 1.07) 0.234 |
| Hypertension |  |  |  |  |
| No | 7,238 | 1 | 0.86 (0.71, 1.03) 0.101 | 0.79 (0.65, 0.96) 0.018 |
| Yes | 8,413 | 1 | 0.92 (0.83, 1.02) 0.095 | 0.83 (0.75, 0.93) 0.001 |
| Coronary heart disease |  |  |  |  |
| Yes | 973 | 1 | 0.84 (0.66, 1.08) 0.168 | 0.79 (0.62, 1.02) 0.067 |
| No | 14,678 | 1 | 0.91 (0.83, 1.00) 0.062 | 0.83 (0.75, 0.92) <0.001 |
| Stroke |  |  |  |  |
| Yes | 754 | 1 | 1.07 (0.82, 1.40) 0.603 | 0.80 (0.60, 1.08) 0.151 |
| No | 14,897 | 1 | 0.88 (0.80, 0.96) 0.007 | 0.82 (0.74, 0.90) <0.001 |

Each stratification adjusted for all the factors (age, gender, race, education, BMI, drinking status, smoking status, diabetes, hypertension, coronary heart disease, stroke), except the stratification factor itself.

*BMI, Body mass index.
